# Ileal Signet Ring Cell Carcinoma Masked by Crohn Disease

**DOI:** 10.31486/toj.19.0066

**Published:** 2020

**Authors:** Muhammad Baraa Hammami, Reem Aboushaar, Ahmad Musmar, Mishah Azhar

**Affiliations:** ^1^ Department of Internal Medicine, Florida Atlantic University, Charles E. Schmidt College of Medicine, Boca Raton Regional Hospital, Boca Raton, FL; ^2^ Florida Atlantic University, Charles E. Schmidt College of Medicine, Boca Raton, FL

**Keywords:** *Carcinoma–signet ring cell*, *Crohn disease*, *ileal neoplasms*, *inflammation*, *intestine–small*

## Abstract

**Background:** Signet ring cell carcinoma (SRCC) is a rare, highly malignant adenocarcinoma that generally involves the stomach; ileal involvement is uncommon. Crohn disease (CD) is associated with long-standing inflammation that may predispose to small intestine adenocarcinoma.

**Case Report:** A 67-year-old male with ileal CD since age 23 years, maintained in remission by mesalamine, presented with mild intermittent attacks of abdominal cramping, an increase in bowel movements from 3 to 5 daily, and bloating for 3 months. Computed tomography enterography with contrast enhancement demonstrated 2 segments of ileal wall thickening. Colonoscopy performed 7 years prior was unremarkable. The patient received oral prednisone with mild symptomatic improvement; he declined biologics. Ileocolonoscopy 1 month later revealed a nontraversable terminal ileal stricture 15 cm from the ileocecal valve. Biopsy demonstrated signet ring cells infiltrating the lamina propria. The patient underwent laparoscopic ileocecectomy and ileocolic anastomosis. Histopathology of a 2.5-cm ileal mass showed poorly differentiated adenocarcinoma with mucin production and signet ring cell features. One metastatic mesenteric lymph node was identified. Adjuvant chemotherapy was initiated.

**Conclusion:** This case of metastatic ileal SRCC occurred in the setting of long-standing, clinically controlled CD. Although the absolute risk of small-bowel adenocarcinoma in CD is low, active surveillance for small-bowel adenocarcinoma in patients with longstanding CD may be prudent, given the overlapping symptomology of SRCC and CD, the aggressiveness of SRCC, and the association of SRCC with subclinical inflammation.

## INTRODUCTION

Small-bowel malignancies are rare, accounting for 3% of gastrointestinal tract neoplasms.^1^ Adenocarcinomas represent 25% to 40% of small-bowel neoplasms.^2^ Signet ring cell carcinoma (SRCC) is a rare adenocarcinoma that generally involves the stomach but can involve other organs, including the small intestine.^3^ SRCC is poorly differentiated and has a poor prognosis.^4,5^

Crohn disease (CD) is a well-known risk factor for intestinal cancer,^3^ arguably because of CD-associated inflammation.^6^ We report a case of ileal SRCC in a patient with long-standing, clinically controlled CD.

## CASE REPORT

A 67-year-old male with ileal CD since age 23 years, maintained in remission by mesalamine, presented with mild intermittent attacks of abdominal cramping, an increase in bowel movements from 3 to 5 daily, and bloating for 3 months. Abdominal examination revealed mildly diffuse tenderness with hyperactive bowel sounds. Leukocyte count was 8.3 cells/μL, erythrocyte sedimentation rate was 33 mm/h, and C-reactive protein was 5.6 mg/L.

**Figure 1.**
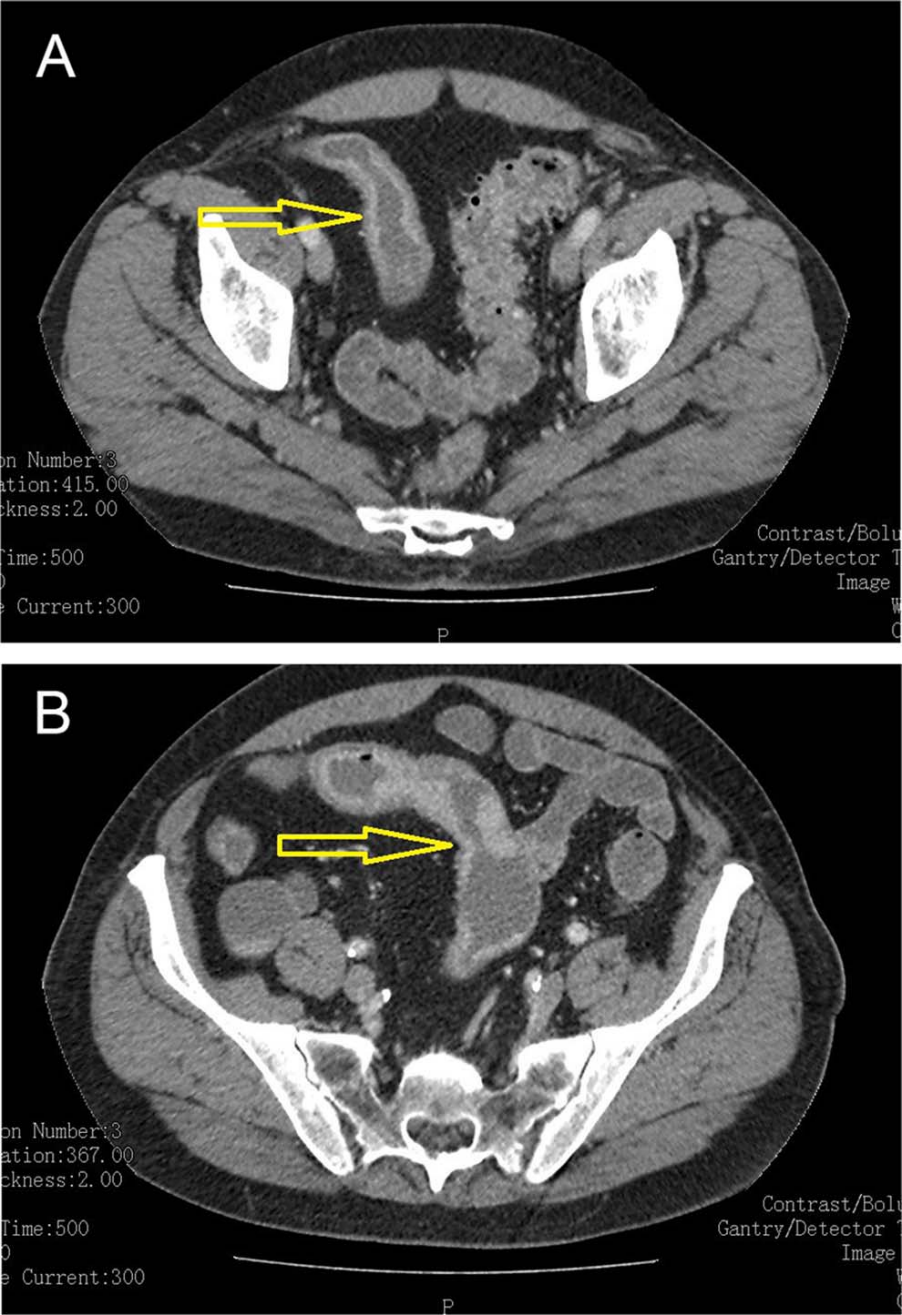
**(A and B) Axial section of computed tomography enterography demonstrates wall thickening of several loops of the ileum (arrows).**

Computed tomography (CT) enterography with contrast enhancement demonstrated 2 segments of ileal wall thickening (Figure 1). Colonoscopy performed 7 years prior was unremarkable. The patient took oral prednisone 40 mg/day for 1 week, followed by gradual tapering for 1 month for suspected partial inflammatory small-bowel obstruction. He reported mild symptomatic improvement. The patient declined biologics.

**Figure 2.**
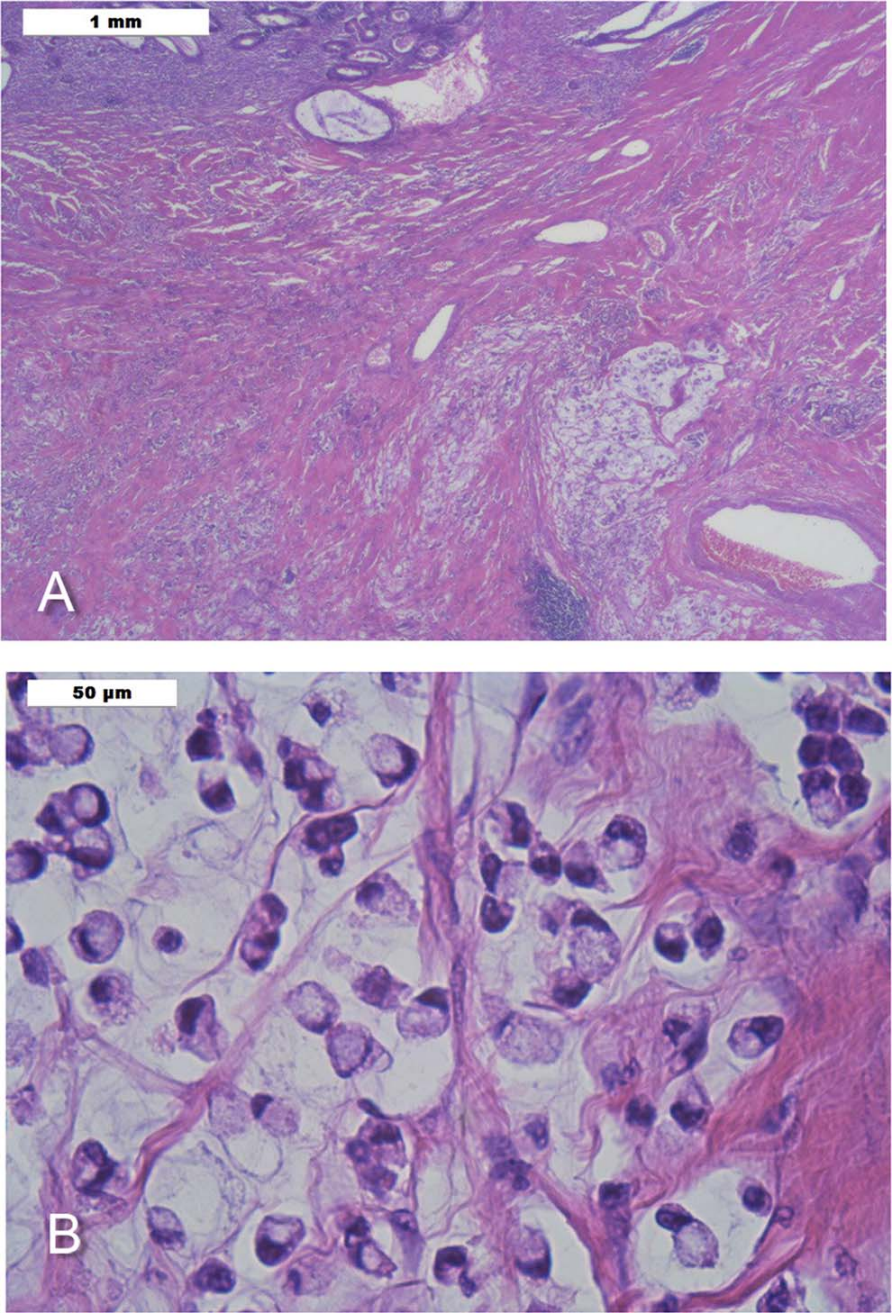
**Histopathologic examination of a 2.5-cm ileal mass demonstrates (A) mucin-producing poorly differentiated adenocarcinoma in muscularis propria and (B) poorly differentiated adenocarcinoma with signet ring cell features.**

Ileocolonoscopy 1 month later revealed a nontraversable terminal ileal stricture 15 cm from the ileocecal valve. Biopsy demonstrated signet ring cells infiltrating the lamina propria. The patient underwent laparoscopic ileocecectomy and ileocolic anastomosis. Histopathology of a 2.5-cm ileal mass showed poorly differentiated adenocarcinoma with mucin production and signet ring cell features (Figure 2), a 6-cm tubulovillous adenoma, and active CD stricturing. Cytokeratin 20 and caudal-type homeobox transcription factor 2 immunostains were positive. One metastatic mesenteric lymph node was identified. Whole-body CT scan was otherwise negative. Upper endoscopy and stomach biopsy were negative for malignancy. Positron emission tomography scan was unremarkable.

Adjuvant chemotherapy with FOLFOX regimen (folinic acid, fluorouracil, and oxaliplatin) was initiated, but follow-up information was not available because the patient moved to another state.

## DISCUSSION

Ileal SRCC in patients with CD is extremely rare. A review of the literature yielded 8 cases.^3,7-13^ Including our patient, the mean age was 50.9 years (range, 31 to 67 years), 55% were female, 89% presented with abdominal pain, and the mean CD duration (duration was not reported in 1 case^13^) was 20.4 years (range, 0 to 44 years). One patient had a history of right ileocolectomy for intestinal obstruction from CD before the SRCC diagnosis.^9^

Patients with CD are thought to be at higher risk of small-bowel adenocarcinoma compared to the general population because of CD-associated inflammation.^6^ Palascak-Juif et al reported the cumulative risk of small-bowel adenocarcinoma to be 0.2% at 10 years for patients with small-bowel CD.^14^ Thus, if *long-standing* is defined as ≥10 years, most of the patients in the reported cases had long-standing CD. The CD duration was 10 to 44 years in our case and in 5 of the other cases,^7,9-12^ was 7 years in 1 case,^8^ and was unreported in 1 case.^13^ One patient received a simultaneous diagnosis of CD and ileal SRCC.^3^

Clinically, our patient had relatively well-controlled disease, suggesting that even subclinical inflammation may be contributory and that aggressive medical therapy (eg, biologic agents such as anti–tumor necrosis factor-alpha, anti-integrins, anti–interleukin-12, and anti–interleukin-23) and close surveillance may be beneficial, even in the presence of symptomatic remission. In our patient, the overlap in symptomology between SRCC and CD led to delayed diagnosis.

## CONCLUSION

Although the absolute risk of small-bowel adenocarcinoma in CD is low, active surveillance for small-bowel adenocarcinoma in patients with long-standing CD may be prudent, given the overlapping symptomology of SRCC and CD, the aggressiveness of SRCC, and the association of SRCC with subclinical inflammation.

## Notes

This article meets the Accreditation Council for Graduate Medical Education and the American Board of Medical Specialties Maintenance of Certification competencies for Patient Care and Medical Knowledge.
